# Tunes that move us: the impact of music-induced emotions on prosocial decision-making

**DOI:** 10.3389/fpsyg.2024.1453808

**Published:** 2025-01-09

**Authors:** Hongwei Wu, Danni Wang, Linshu Zhou

**Affiliations:** ^1^School of Music and Dance, Communication University of Zhejiang, Hangzhou, China; ^2^Music College, Shanghai Normal University, Shanghai, China

**Keywords:** music, prosocial behavior, mood and arousal, empathy, theory of mind, music mode and tempo

## Abstract

**Introduction:**

The significance of music might be attributed to its role in social bonding, a function that has likely influenced the evolution of human musicality. Although there is substantial evidence for the relationship between prosocial songs and prosocial behavior, it remains unclear whether music alone, independent of lyrics, can influence prosocial behaviors. This study investigates whether music and the emotions it induces can influence prosocial decision-making, utilizing the classical two-dimensional model of emotion (mood and arousal).

**Methods:**

In Experiment 1,42 undergraduate students listened to happy music (positive, high arousal), sad music (negative, low arousal), and white noise while reading stories describing helping scenarios and then assessed their willingness to help. Experiments 2 and 3 further explore mood and arousal effects by manipulating the mode (major vs. minor) and tempo (fast vs. slow) of the music.

**Results:**

Experiment 1’s results indicated that sad music increases willingness to help more than happy music or white noise, suggesting that music-induced emotions influence prosocial behavior through immediate prosocial emotions like empathy. Experiments 2 and 3 demonstrated that only mood, influenced by the music mode, affects prosocial decision-making, while tempo-induced arousal does not. Additionally, Theory of Mind and memory strength do not mediate these effects.

**Discussion:**

These findings reveal the role of pure music listening and specific emotional dimensions on prosocial decision-making, providing evidence to support the music-social bonding hypothesis.

## 1 Introduction

Music, a product of human consciousness, permeates every known human culture (Mehr et al., 2019). Its evolutionary origins remain a subject of debate, but its significance is likely rooted in its contribution to human evolution (Darwin, 1871; Wallin et al., 2001). As inherently social creatures, humans depend on interactions and cooperation for survival and development. It has been suggested that music plays a vital role in enhancing social bonds throughout human evolution, underscoring its evolutionary importance (Cross, 2001; Fukui and Toyoshima, 2023; Huron, 2001; Savage et al., 2021; Weinstein et al., 2016). Given that prosocial behaviors—such as helping, volunteering, and sharing—are crucial indicators of social connections, research exploring how music promotes prosocial decision-making is essential for confirming its functional value in social bonding.

Research has indicated that prosocial songs, which contain lyrics promoting social cooperation, can significantly influence behavior. Greitemeyer (2009b) reveals that listeners of prosocial songs are more likely to engage in unpaid research and demonstrate increased cooperative behaviors than those who listen to neutral songs. Further investigations support these results, demonstrating that such listeners tend to make greater charitable donations (Greitemeyer, 2009a; Ruth and Schramm, 2021), tip more generously (Jacob et al., 2010), and purchase more environmentally friendly coffee (Ruth, 2017). Moreover, prosocial music encourages proactive behaviors, like picking up dropped pens (Greitemeyer, 2011a; Kennedy, 2013) and distributing more flyers (Greitemeyer and Schwab, 2014). Additionally, prosocial songs have been shown to reduce aggressive behaviors and tendencies (Greitemeyer, 2011b; Greitemeyer and Schwab, 2014; Jacob et al., 2010). These effects are influenced by factors such as empathy (sharing another’s feelings), Theory of Mind (ToM; i.e., inferring others’ mental states), cognitive processes, and music familiarity (Greitemeyer, 2022; Ruth and Schramm, 2021; Ruth, 2019).

Relatively few studies have explored whether music alone, independent of lyrics, has prosocial effects. Some research suggests that instrumental music can also have prosocial effects. Fried and Berkowitz (1979) found that participants who listened to soothing and stimulating music were more willing to help than those who listened to aversive music or remained in silence, indicating that musical emotions can influence prosocial behavior. Contrarily, other studies have suggested that the prosocial impact of pure music may be limited. Ganser and Huda (2010) found no difference in virtual donation amounts between participants who listened to uplifting music and those in a silent condition. A more recent study by Yu et al. (2019) investigated the influence of lyrics and music on prosocial decision-making. Their results indicated that when lyrics had prosocial content, the music accompanying them (i.e., songs) was more effective in promoting prosocial decisions than reading the lyrics without music. However, when the lyrics were neutral, the presence or absence of music did not affect prosocial decision-making. This finding suggests that the prosocial effect of the music in songs may depend on them. Therefore, the evidence on whether music alone can have prosocial effects, excluding the influence of lyrics, remains inconsistent.

Compared to language, music is also a medium for expressing emotions and meaning (Meyer, 1956; Patel, 2008; Zhou et al., 2014, 2015). The emotional power of music is well-documented, with studies showing that it can evoke a wide range of emotions (Juslin and Västfjäll, 2008; Konečni, 2008), though the underlying mechanisms are still being explored (e.g., Huron, 2006; Juslin, 2013; Koelsch, 2010, 2014). Some evidence suggests that music-induced emotions affect risk-taking behaviors (Halko et al., 2015; Schulreich et al., 2014) and intertemporal decision-making (Zhou et al., 2022). However, the relationship between music-induced emotions and prosocial behavior is not fully understood. Although some studies have explored the link between music-induced mood and arousal and prosocial decision-making (Beer and Greitemeyer, 2019; Fried and Berkowitz, 1979; Fukui and Toyoshima, 2014; Ganser and Huda, 2010; Kennedy, 2013; McDonald et al., 2022), different musical pieces across conditions might introduce confounding acoustic and structural factors.

The two-dimensional model of emotion (mood and arousal) provides a useful framework for understanding the potential effect of music-induced emotions on prosocial behavior (Russell, 1980). Music can elicit high-pleasure, high-arousal emotions like happiness or low-pleasure, low-arousal emotions like sadness, each affecting behavior differently (Palazzi et al., 2019). Happiness and sadness are the most reliable and distinct musically induced emotions (Balkwill and Thompson, 1999). In Western tonal music, these emotions are shaped by two key features: mode (major or minor) and tempo (speed of the beat) (Krumhansl, 1997). Major mode music with a fast tempo evokes happiness, while minor mode music with a slow tempo evokes sadness (Peretz et al., 1998). Thus, mode and tempo explain the effects of mood and arousal dimensions, with major music being more pleasant than minor music, and fast music more arousing than slow music. Manipulating mode and tempo can help disentangle the effects of mood and arousal on prosocial behavior, clarifying the role of specific acoustic and structural factors in music.

Furthermore, empathy is a crucial concept in understanding the social and emotional impact of musical experiences (Clarke et al., 2015; Miu and Vuoskoski, 2017). Empathy refers to the ability or psychological process by which individuals accurately perceive, experience, and resonate with the emotional states of others (Preston and de Waal, 2002). It plays a crucial role in social interaction and cooperation (Han et al., 2009) and drives prosocial behavior, prompting actions that benefit others (Batson et al., 2007; Eisenberg and Miller, 1987; Roberts and Strayer, 1996). Empathy is both a stable trait and a dynamic process; trait empathy is a consistent ability, while state empathy involves real-time emotional responses (Cuff et al., 2016). It also comprises two components: cognitive empathy, which involves understanding others’ emotions and perspectives, and affective empathy, which involves sharing their emotional states (Davis, 1983; Walter, 2012). Importantly, it has been suggested that empathy is intricately involved in the emotional responses induced by music (e.g., Clarke et al., 2015; Juslin, 2013; Juslin and Västfjäll, 2008; Livingstone and Thompson, 2009; Scherer and Zentner, 2001). For instance, Egermann and McAdams (2013) demonstrated that an individual’s level of empathy moderates both perceived and induced emotions from music. Moreover, empathy not only influences physiological reactions to music (Miu and Balteş, 2012) but has also been associated with a greater susceptibility to sadness induced by music (Vuoskoski and Eerola, 2012). Empathic individuals tend to derive more enjoyment from sad music compared to non-empathic individuals, suggesting that empathically experienced negative emotions, such as sadness, can be enjoyable in the context of music (Garrido and Schubert, 2011; Greenberg et al., 2015; Vuoskoski et al., 2012). Individuals with high levels of empathy are better at recognizing emotions expressed in music, particularly sadness (Eeroia et al., 2016; Kawakami and Katahira, 2015; Wöllner, 2012). The connection between empathy and music-induced sadness implies that sad music might promote prosocial behavior.

The present study aimed to examine the impact of music-induced emotions on prosocial decision-making through three experiments, each systematically exploring the distinct effects of different musical features on prosocial behavior. First, Experiment 1 investigated the influence of music-induced happiness and sadness on prosocial decision-making by using happy (fast major) music, sad (slow minor) music, and white noise (control condition) to induce the corresponding emotions. Participants read helping stories and then assessed their willingness to help. To further distinguish the effects of mood and arousal on prosocial behavior, Experiment 2 focused on manipulating the mode (major vs. minor) of the background music, while Experiment 3 selectively manipulated the tempo (fast vs. slow). Additionally, we examined the mediating effects of memory strength and measured participants’ affective empathy, familiarity with the music, and musical preferences to investigate related mechanisms and factors. Given the role of ToM in understanding and predicting others’ behavior, this study also explored its mediating role, specifically the ability to infer others’ mental states. We expected that music-induced emotions would influence prosocial decision-making, with mode and its effect on mood playing a significant role.

## 2 Experiment 1: effect of happy and sad music on prosocial decision-making

As an initial test of the effect of music-induced emotions on prosocial decision-making, we manipulated the happy-sad emotions of the background music while imagining a helping episode. We set the imagined helping events in either happy (i.e., fast major music), sad (i.e., slow minor music) or neutral emotions (i.e., white noise). We hypothesized that imagining helping events with sad background music would increase one’s willingness to help compared to imagining events with happy background music, as a direct result of the increased sad emotions induced by imagining a helping event scene in the context of sad music.

### 2.1 Materials and methods

#### 2.1.1 Participants

We applied a within-subjects design in which all participants listened to happy music, sad music, and white noise. *A priori* power analysis using G-power 3.1 (Faul et al., 2009) determined that, to achieve a statistical power of 80% for a repeated measures ANOVA *F*-test detecting a medium effect (*f* = 0.25), at least 36 participants were needed. Consequently, 42 university students (31 females, 11 males) aged 19 to 29 (*M* = 22.83, *SD* = 2.11) participated in the experiment. Aside from general music education in primary and secondary school, no participants had received more than 3 years of extracurricular music training. All participants provided informed written consent before the experiment and were compensated for participating afterward.

#### 2.1.2 Materials

##### 2.1.2.1 Musical stimuli

The musical stimuli in this experiment consisted of 14 piano excerpts selected from a well-established set of musical stimuli designed to evoke specific emotions (Peretz et al., 1998). This set has been extensively used in music and emotion research due to its robust design and validated emotional content, and it can be accessed online at https://peretzlab.ca/online-test-material/material/mode-tempo/. All stimuli were MIDI-based, meaning they were created using digital encoding of musical information rather than pre-recorded (sampled) audio tracks. This approach allows precise control over musical parameters and ensures consistency across experimental conditions. Furthermore, the stimuli were synthesized rather than humanly performed, which reduces potential variability in performance nuances and maintains a consistent auditory presentation for all participants. Although performance expressiveness contributes to the emotional content of music, when manipulating general features such as mode or tempo, controlling for performance nuance by using mechanically synthesized MIDI stimuli seems warranted to reduce unanticipated interactions.

Detailed information about the 14 excerpts used in this experiment is provided in Supplementary Table S1. These pieces, composed between 1700 and 1900, represent Baroque, Classical, Romantic, and Contemporary Western tonal music. Of these, seven pieces expressed happiness and seven expressed sadness. The happy music was characterized by major keys and fast tempos (80 ~ 255 BPM), while the sad music was characterized by minor keys and slow tempos (20 ~ 100 BPM). Additionally, seven white noise tracks were used as a control condition, selected from a previous study (Zhou et al., 2022) due to their relatively neutral emotional characteristics (Nyklíček et al., 1997). All stimuli were edited using Adobe Audition CS6 software (Adobe Systems Incorporated, San Jose, California, United States) at a resolution of 44.1 kHz, 16-bit, and had an average duration of 30 s. The sound levels were standardized using Adobe Audition CS6 to approximately 68 dB SPL.

##### 2.1.2.2 Stories

Initially, 27 stories were written, each depicting a person in need. These stories comprised one paragraph containing six long sentences, with an average reading time of about 30 s. For example: “*周五晚上，王妍加完班后准备回家。(On Friday night, after working overtime, Wang Yan was ready to go home.) 她赶往剬交车站，搭乘上了最后一班18路剬交车。(She hurried to the bus stop and caught the last Route 18 bus.) 她走上剬交车，量了体温后，从包里掏出手机。(After boarding, she had her temperature checked and pulled her phone out of her bag.) 她打开电子乘车卡的页面，此时，手机自动关机了。(Just as she opened the electronic bus card page, her phone suddenly powered off.)王妍重启手机也没反应，她到包里翻了翻，也没有零钱。(Wang Yan tried to restart her phone, but it did not respond. She searched through her bag but could not find any loose change.)她站在前边车门处，不知如何是好。(She stood by the front door of the bus, not knowing what to do.)*”.

To assess the effectiveness of the story materials, 7 participants were recruited for a pre-test before the formal experiment. These participants did not take part in any of the formal experiments. During the pre-test, participants were asked to read all 27 texts and evaluate the comprehensibility of the materials, their emotional experience after reading, the extent to which the protagonist needed help, and the cost of providing help. The comprehensibility of the texts was assessed using a 7-point scale (7 = very easy to understand to 1 = very difficult to understand). Emotional responses were evaluated using a 7-point scale (7 = very happy to 1 = very sad). The extent to which help was needed was assessed using a 4-point scale (4 = much help needed to 1 = no help needed). The cost of providing help was also evaluated using a 4-point scale (4 = high cost to 1 = no cost).

Finally, 21 stories were selected as the formal materials based on the pre-test results. The chosen stories were highly comprehensible (*M* = 6.82, *SD* = 0.22), emotionally neutral (*M* = 3.56, *SD* = 0.36), with protagonists moderately in need of help (*M* = 2.86, *SD* = 0.51), and with moderate help costs (*M* = 2.14, *SD* = 0.46). All story materials used in the formal experiment can be found in Supplementary Table S2.

#### 2.1.3 Procedure

The experiment was conducted in a quiet room, with the experimental procedure presented via computer. At the beginning of the experiment, each participant was asked to complete an initial assessment of their emotional state to establish a baseline level. This assessment included three aspects: the experience of happy–sad emotions and the dimensions of mood and arousal. Participants used a 7-point scale to rate their current feelings on a happiness-sadness continuum (7 = very happy to 1 = very sad). Then they rated their mood (7 = very pleasant to 1 = very unpleasant) and arousal (7 = highest arousal to 1 = lowest arousal) on 7-point scales.

After assessing their baseline emotional state, participants read 21 stories. Each story was presented visually on the computer screen in text form for 30 s. To create an immersive reading experience and enhance focus, the text of each story was presented word by word over time rather than all at once. The participants were instructed to imagine the story’s scenario and jot down any related words, thoughts, or feelings on paper to ensure they paid close attention to each story. Notably, during the reading of each story, participants listened to 30 s of music played as background sound through headphones. Each story was presented with background music in three emotional emotions: happy, sad, or neutral. To ensure each story and music stimulus appeared only once for each participant, we used a Latin square design to create three lists. In each list, every story was presented under a single music condition. Each list contained 21 stories, with seven stories for each music condition. The stimuli were presented in random order within each list. During the experiment, the three lists were equally distributed across the 42 participants. After reading each story, participants reassessed their emotional experiences using the same 7-point scales for happy–sad emotions, mood, and arousal. This assessment took at least 10 s before they could proceed to the next story.

After reading all 21 stories with background music and completing 21 emotional state assessments, the stories were presented again in the same order but without background music or sound. Participants then evaluated their willingness to help the protagonist (7 = very willing to 1 = very unwilling) and their ToM (7 = strongly considered the protagonist’s thoughts and feelings to 1 = did not consider them at all). Given that memory strength can reflect cognitive ability—the stronger the memory, the stronger the cognitive ability—the experiment also assessed participants’ memory strength for each story using a 5-point scale (5 = strongly agree to 1 = strongly disagree with “I can quickly recall this story upon re-reading”). After completing all story evaluations, participants’ trait empathy was assessed using the empathic concern subscale of the Interpersonal Reactivity Index (Davis, 1983).

To examine the influence of music preference and familiarity on the experimental results, a post-test was conducted at the end of the experiment. Participants rated their preference and familiarity with the music in the order presented. Preference was rated on a 7-point scale (7 = very much liked to 1 = very much disliked). Familiarity was assessed as a yes-or-no question; if the music or sound was familiar, participants provided the title or composer’s name.

#### 2.1.4 Statistical analyses

Firstly, the experiment analyzed the differences in participants’ emotional states under different music conditions to determine whether the emotional induction effect of the music was successful. Repeated measures ANOVAs or paired samples *t*-tests were employed to compare emotional states across music conditions. When significant differences were found, *post hoc* tests were conducted to ascertain significant differences between each pair of conditions. All pairwise comparisons were adjusted by Holm correction. When the degree of freedom in the numerator was larger than one, Greenhouse–Geisser correction was applied. In these cases, the original degrees of freedom with corrected *p* values are reported.

Then, the experiment investigated whether the experience of musical emotions influenced prosocial decision-making. Repeated measures ANOVAs or paired samples *t*-tests were used to compare participants’ willingness to help under different music conditions.

To obtain more conservative results and assess the strength of evidence, we conducted a Bayesian ANOVA analysis and calculated Bayes factors (*BF*s) using JASP software (van Doorn et al., 2021) with the default priors. The *BF* represents the ratio of the probability of one hypothesis over another, quantifying the relative strength of evidence for the alternative (*H_1_*) and null (*H_0_*) hypotheses (Brydges et al., 2020; Love et al., 2019; Wagenmakers et al., 2018). For instance, a *BF* of 30 indicates that the data are 30 times more likely under *H_1_* than under *H_0_*, providing strong evidence in support of an effect. Generally, 1 < *BF* < 3 is considered weak evidence for *H_1_*, 3 < *BF* < 10 indicates moderate evidence, and *BF* > 10 indicates strong evidence. Conversely, 0.33 < *BF* < 1 indicates weak evidence for *H_0_*, 0.10 < *BF* < 0.33 indicates moderate evidence, and *BF* < 0.10 indicates strong evidence (Jeffreys, 1961; Lee and Wagenmakers, 2014).

To test whether the effect of music-induced emotions on prosocial decision-making was mediated by ToM and memory strength, a bootstrapping procedure was applied for the mediation analysis (Hayes and Preacher, 2014). The independent variable was the type of music and its evoked emotions, while the dependent variable was participants’ prosocial willingness. ToM and memory strength served as mediator variables. The analysis was conducted using PROCESS macro in JASP. The bootstrapping process involved 5,000 resamples, and the statistical significance of indirect paths was determined by 95% confidence intervals (CIs). When 0 was not within the 95% CI, it indicated a significant difference observed in the mediation test.

### 2.2 Results and discussion

#### 2.2.1 Emotional effects induced by music

**Figure 1:**
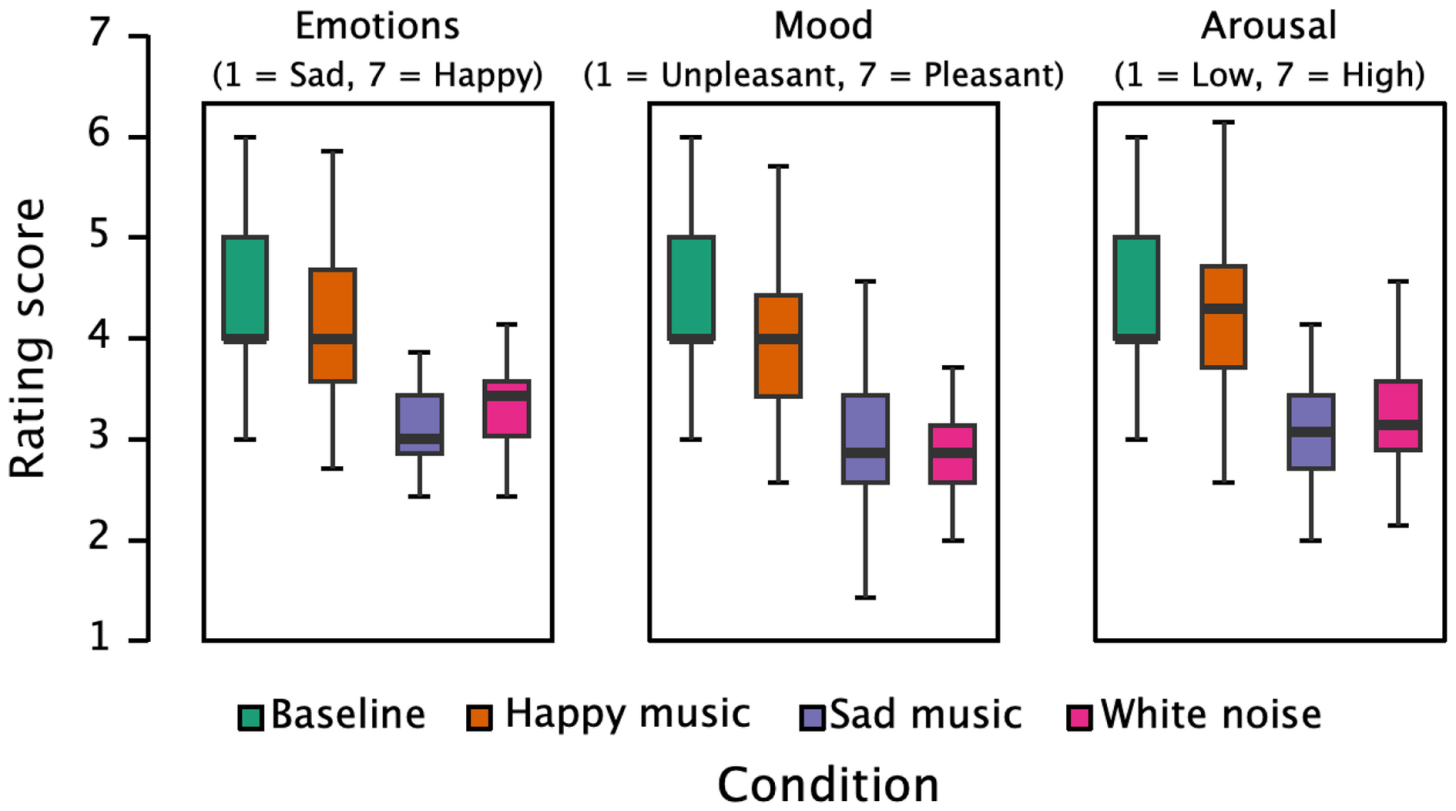
Subjective ratings of emotions (happy/sad), mood, and arousal under baseline and three music conditions.

Figure 1 presents the participants’ ratings across different conditions concerning happy–sad emotions, mood, and arousal. The results of the repeated measures ANOVA indicate strong evidence for significant differences in happy–sad emotion ratings (*F*(3, 123) = 49.899, *p* < 0.001, partial η^2^ = 0.549, *BF* = 4.921 × 10^+18^). Pairwise comparisons revealed that the baseline condition (*M* = 4.45, *SD* = 0.94) had significantly higher ratings compared to the happy music condition (*M* = 4.11, *SD* = 0.74, *p* = 0.013), the sad music condition (*M* = 3.12, *SD* = 0.52, *p* < 0.001), and the white noise condition (*M* = 3.37, *SD* = 0.52, *p* < 0.001). Ratings in the happy music condition were higher than those in both the sad music and white noise conditions (*p*s < 0.001), and white noise elicited higher ratings than sad music (*p* = 0.049). These findings suggest that different types of music induced varying emotional experiences, with sad music eliciting more sadness than happy music and white noise, while white noise resulted in a relatively neutral emotional experience.

Significant differences were also observed in participants’ mood ratings (*F*(3, 123) = 53.884, *p* < 0.001, partial η^2^ = 0.568, *BF* = 2.252 × 10^+20^). Pairwise comparisons indicated that the baseline condition (*M* = 4.55, *SD* = 1.15) had significantly higher pleasure ratings than the happy music (*M* = 3.96, *SD* = 0.81, *p* < 0.001), sad music (*M* = 2.94, *SD* = 0.66, *p* < 0.001), and white noise conditions (*M* = 2.88, *SD* = 0.61, *p* < 0.001). The happy music condition resulted in higher pleasure ratings than sad music and white noise (*p*s < 0.001). However, there was no significant difference in pleasure ratings between the sad music and white noise conditions (*p* = 0.697). These results indicate that music induced different levels of pleasure experiences, with happy music eliciting more pleasure than sad music and white noise.

Furthermore, there were significant differences in participants’ arousal ratings (*F*(3, 123) = 51.074, *p* < 0.001, partial η^2^ = 0.555, *BF* = 6.316 × 10^+19^). Pairwise comparisons showed that the baseline condition (*M* = 4.41, *SD* = 0.91) had significantly higher arousal ratings than the sad music (*M* = 3.08, *SD* = 0.63, *p* < 0.001) and white noise conditions (*M* = 3.20, *SD* = 0.54, *p* < 0.001), while there was no difference between the baseline and happy music conditions (*M* = 4.27, *SD* = 0.72, *p* = 0.624). The happy music condition had higher arousal ratings than both the sad music and white noise conditions (*p* < 0.001), with no difference between the sad music and white noise conditions (*p* = 0.624). These findings indicate that happy music induced higher levels of arousal compared to sad music and white noise.

Additionally, post-test results indicated significant differences in music preference among the three conditions (*F*(2, 82) = 63.338, *p* < 0.001, partial η^2^ = 0.607, *BF* = 2.876 × 10^+17^). Specifically, participants preferred happy music over sad music and white noise (*p* < 0.001), and sad music over white noise (*p* < 0.001). Despite these preferences, all participants reported unfamiliarity with all the music pieces and were unable to correctly identify the titles and composers during the familiarity assessment.

#### 2.2.2 Effects on prosocial decision-making

**Figure 2:**
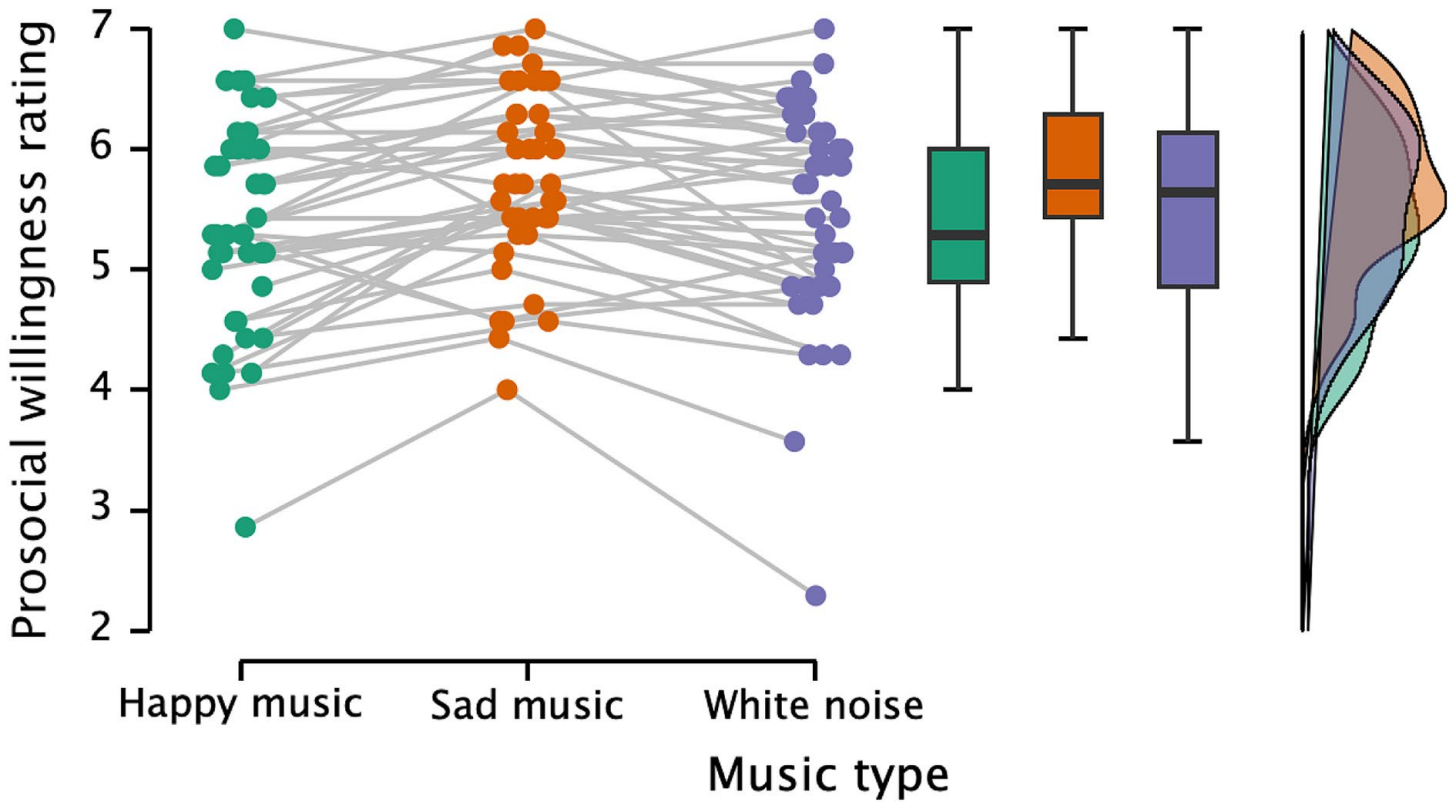
Participants’ ratings of willingness to help under happy music, sad music, and white noise conditions.

Figure 2 shows the participants’ prosocial willingness ratings under different music conditions. A repeated measures ANOVA was conducted with music as the independent variable and willingness to help as the dependent variable. The results indicate strong evidence for a significant effect of music (*F*(2, 82) = 12.136, *p* < 0.001, partial η^2^ = 0.228, *BF* = 783.867). Participants’ willingness to help was significantly higher in the sad music condition (*M* = 5.75, *SD* = 0.73) compared to the happy music condition (*M* = 5.36, *SD* = 0.87, *p* < 0.001). Additionally, participants in the sad music condition showed higher prosocial willingness than those in the white noise condition (*M* = 5.46, *SD* = 0.92, *p* = 0.002). However, there was no significant difference in willingness to help between the happy music and white noise conditions (*p* = 0.195). This suggests that listening to sad music increases participants’ willingness to help compared to listening to happy music and white noise. Additionally, correlation analysis between empathy scores and willingness to help showed no significant relationship under the three music conditions (*N* = 42, *p*s > 0.468).

#### 2.2.3 Mediation analysis

Repeated measures ANOVA revealed differences in ToM scores across the three music conditions (*F*(2, 82) = 4.335, *p* = 0.016, partial η^2^ = 0.096, *BF* = 2.472). Pairwise comparisons showed that participants had significantly higher ToM scores in the sad music condition (*M* = 5.51, *SD* = 0.70) compared to the happy music condition (*M* = 5.31, *SD* = 0.68, *p* = 0.021), and a trend towards higher scores compared to the white noise condition (*M* = 5.35, *SD* = 0.056). This indicates that participants considered the protagonist’s thoughts and feelings more in the sad music condition. There were no differences in memory strength across the three conditions (*F*(2, 82) = 0.321, *p* = 0.726, partial η^2^ = 0.008, *BF* = 0.099).

A mediation model was constructed with music as the independent variable, willingness to help as the dependent variable, and ToM and memory strength as mediating variables. The results indicated that participants were more willing to help in the sad music condition than the happy music condition (*B* = 0.388, *SE* = 0.175, *p* = 0.027, 95% CI [0.039, 0.722]). However, neither ToM nor memory strength mediated the relationship between music-induced emotions and prosocial decision-making (indirect effects: ToM, *B* = 0.151, *SE* = 0.120, 95% CI [−0.074, 0.404]; memory strength, *B* = −8.802 × 10^−4^, *SE* = 0.006, 95% CI [−0.045, 0.026]).

In alignment with prior research (e.g., Dalla Bella et al., 2001; Khalfa et al., 2005; Peretz and Zatorre, 2005; Zhou et al., 2022), this study supports the effectiveness of happy and sad background music in evoking corresponding emotional responses. Reflecting the findings of Hunter et al. (2010), the current experiment further confirmed that happy emotional experiences are associated with higher arousal and pleasure, while sad emotional experiences correlate with lower arousal and pleasure. Compared to the baseline condition, participants’ ratings of emotion, pleasure, and arousal were lower after listening to music. This may be because participants read helping scenario stories while listening to the music. Background music may intensify participants’ feelings for the protagonist of the story, thus inducing state empathy. Therefore, ratings were lower even after listening to happy music. However, since the main focus was on the differences between happy and sad music, and these differences were significant, the experimental manipulation was effective.

Notably, the experiment revealed that participants exposed to sad music exhibited a significantly greater willingness to help compared to those in the happy music and white noise conditions, highlighting the impact of music-induced emotions on prosocial decision-making. This finding aligns with previous research (Lerner et al., 2015; Small and Lerner, 2008; Yang et al., 2017). For instance, in a study conducted by Small and Lerner (2008), participants who were in a sad mood during welfare assistance simulations recommended that rescuers increase the amount of welfare aid. Although various music conditions impacted participants’ ToM scores, neither ToM nor memory strength mediated the relationship between music-induced emotions and prosocial behavior decisions. This raises questions about which specific features of the music and their emotional effects were responsible for the observed impact on prosocial decision-making. Mode and tempo, with their associated emotional characteristics, could have different influences on prosocial behavior. Therefore, Experiments 2 and 3 were designed to isolate and examine the emotional effects of mode and tempo.

## 3 Experiment 2: effect of mode-induced emotions on prosocial decision-making

To investigate the effects of mode-induced emotions on prosocial decision-making, this experiment built on Experiment 1 by controlling for the influence of tempo to isolate the effects of musical mode and its induced emotions. Specifically, the same musical pieces from Experiment 1 were used, but their tempos were standardized. The originally happy music, while retaining its major mode characteristics, was adjusted to a medium tempo; similarly, the originally sad music, while maintaining its minor mode features, was also adjusted to a medium tempo. This ensured that the emotional experiences induced by the music were primarily attributable to mode rather than tempo.

The hypotheses for this study were as follows: First, it was expected that different musical modes would induce distinct emotional experiences, with major music being more pleasant than minor music (Droit-Volet et al., 2010; Husain et al., 2002). Second, based on the results from Experiment 1, it was hypothesized that participants would exhibit higher prosocial willingness under the minor music condition compared to the major music condition. Finally, it was anticipated that neither ToM nor memory levels would mediate the relationship between music-induced emotions and prosocial decision-making.

### 3.1 Materials and methods

#### 3.1.1 Participants

We applied a within-subjects design, in which all participants listened to major and minor music. *A priori* power analysis using G-power 3.1 (Faul et al., 2009) determined that, to achieve a statistical power of 80% for a paired samples *t*-test detecting a medium effect (*d* = 0.6), at least 24 participants were needed. Consequently, 28 university students (20 females, 8 males) aged 19 to 25 (*M* = 22.36, *SD* = 1.67) participated in the experiment. Aside from general music education in primary and secondary school, none of the participants had received more than 3 years of extracurricular music training. All participants provided informed written consent before the experiment and were compensated for their participation afterward.

#### 3.1.2 Materials and procedure

The stimuli in Experiment 2 consisted of 14 musical excerpts directly selected from the well-established set of stimuli provided by Peretz et al. (1998). These stimuli included the seven happy and seven sad pieces used in Experiment 1. Specifically, the tempos of the 14 musical excerpts from Experiment 1 were electronically modified to a neutralized tempo by adjusting all tempos to the median tempo of the original pieces (quarter note = 84 M.M.). Although the tempos were standardized, the mode (major or minor) of each piece was preserved. Consequently, the final stimuli for this experiment included seven major mode and seven minor mode musical excerpts. Additionally, 14 stories were randomly selected from the 21 stories used in Experiment 1 to serve as the helping scenario materials for this experiment. The experimental procedure and statistical methods were consistent with those used in Experiment 1.

### 3.2 Results and discussion

#### 3.2.1 Emotional effects induced by music

**Figure 3:**
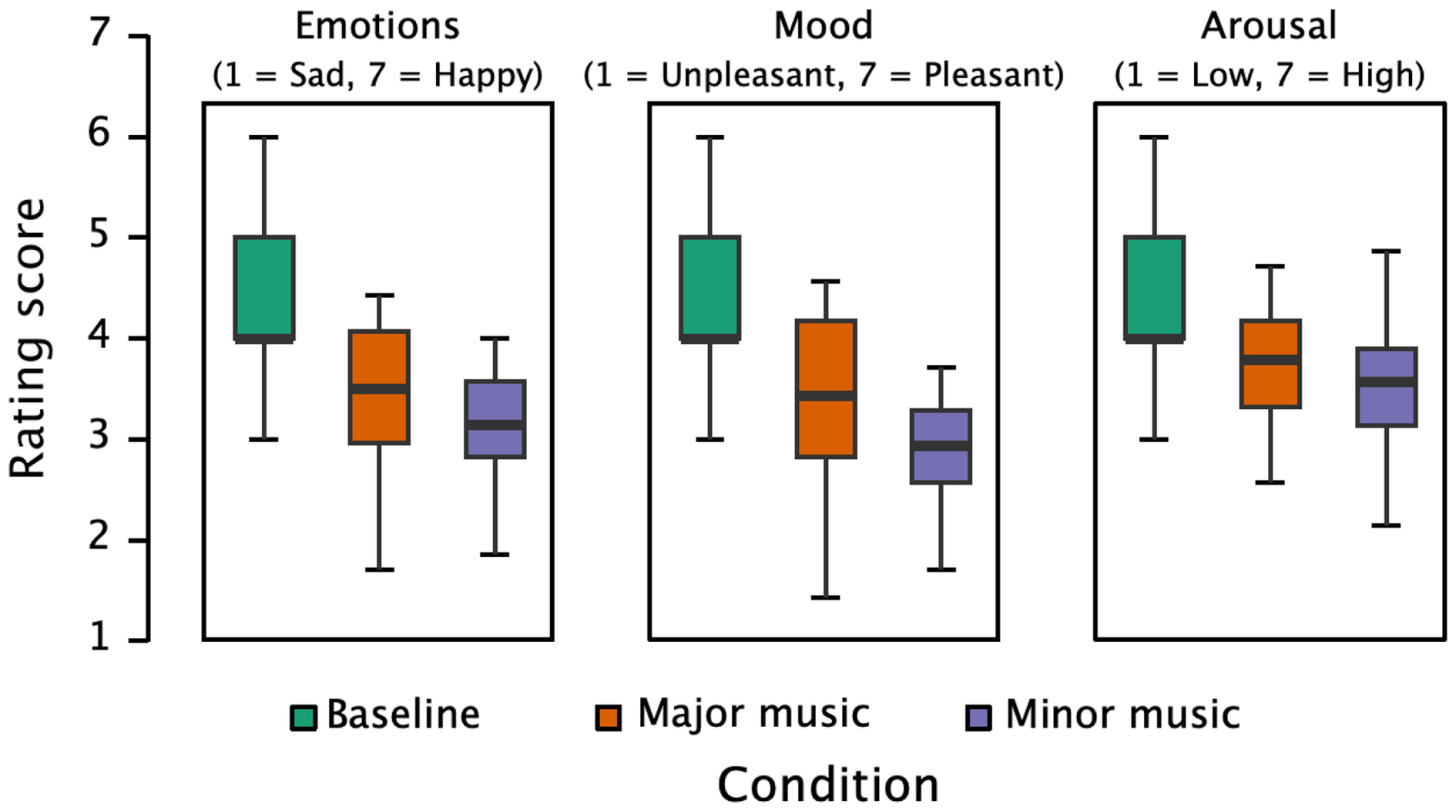
Subjective ratings of emotions (happy/sad), mood, and arousal under baseline, major music, and minor music conditions.

Figure 3 displays participants’ ratings of happy-sad emotions, mood, and arousal under different conditions. There is strong evidence supporting the effect of conditions on happy-sad emotion ratings (*F*(2, 54) = 23.985, *p* < 0.001, partial η^2^ = 0.470, *BF* = 2.124 × 10^+6^). Participants’ ratings on happy-sad emotions in the baseline condition (*M* = 4.25, *SD* = 0.80) were higher than those in both the major (*M* = 3.43, *SD* = 0.79, *p* < 0.001) and the minor music conditions (*M* = 3.09, *SD* = 0.54, *p* < 0.001). The difference between major and minor music conditions was marginally significant (*p* = 0.051).

There is strong evidence supporting the effect of music on mood (*F*(2, 54) = 34.122, *p* < 0.001, partial η^2^ = 0.558, *BF* = 3.187 × 10^+8^). Participants felt more pleasure in the baseline condition (*M* = 4.46, SD = 0.92) than in both the major music condition (*M* = 3.38, *SD* = 0.90, *p* < 0.001) and the minor music condition (*M* = 2.91, *SD* = 0.65, *p* < 0.001). More importantly, mood ratings were significantly lower in the minor music condition than in the major music condition (*p* = 0.019).

For arousal ratings, although participants’ arousal ratings in the baseline condition (*M* = 4.31, *SD* = 0.67) were higher than those in the major music condition (*M* = 3.59, *SD* = 0.83) and the minor music condition (*M* = 3.44, *SD* = 0.69), *F*(2, 54) = 13.636, *p* < 0.001, partial η^2^ = 0.336, *BF* = 4093.396, pairwise comparisons indicated no difference between major and minor music conditions (*p* = 0.413). This suggests that the manipulation of musical modes primarily affects participants’ mood rather than their arousal levels.

Moreover, post-test results showed that participants preferred listening to major music (*M* = 4.85, *SD* = 0.90) over minor music (*M* = 3.98, *SD* = 0.95), *t*(27) = 3.697, *p* < 0.001, Cohen’s *d* = 0.699, *BF* = 34.308. Consistent with Experiment 1, all participants reported unfamiliarity with all the music pieces and were unable to correctly identify the titles and composers during the familiarity assessment.

#### 3.2.2 Effects on prosocial decision-making

**Figure 4:**
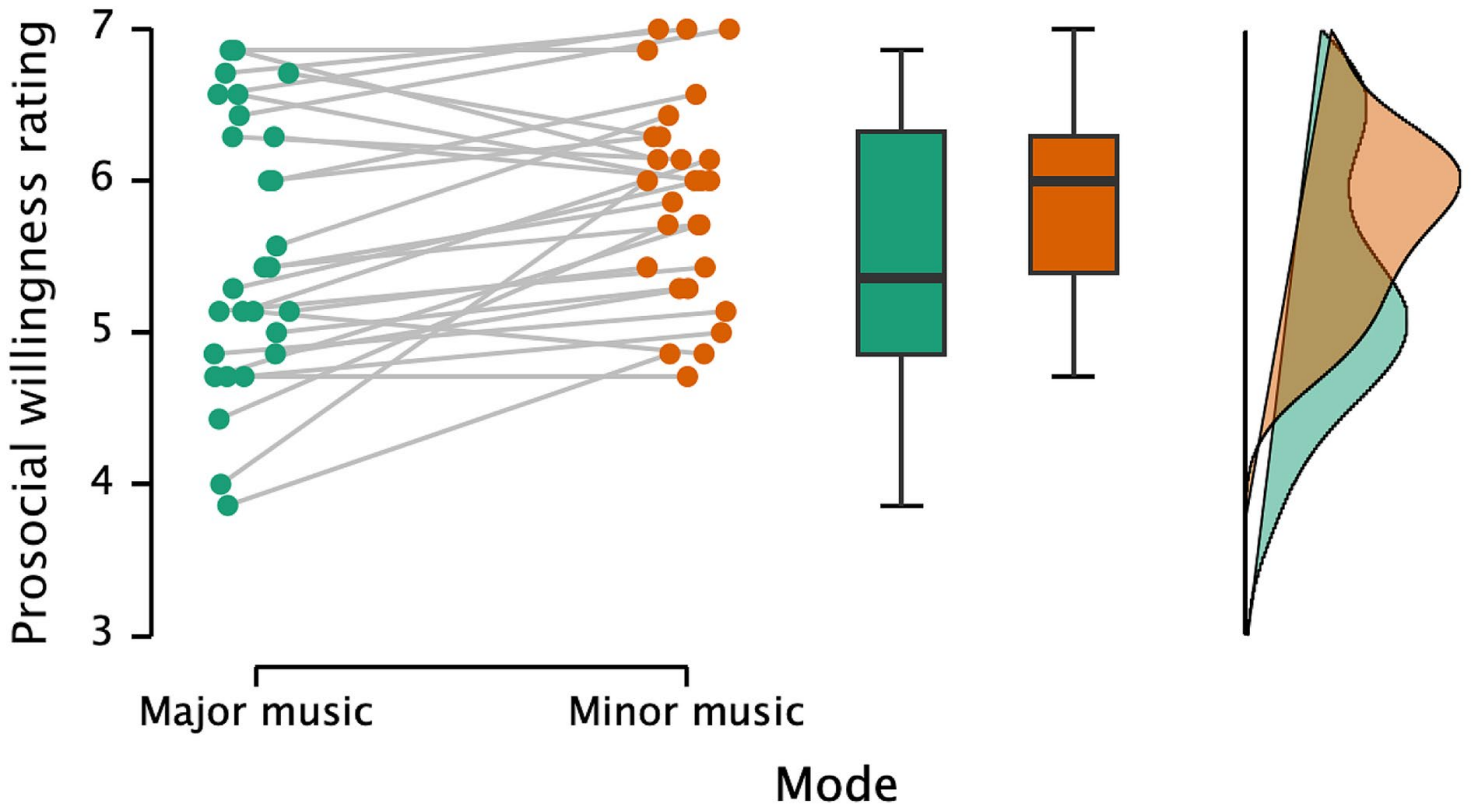
Participants’ ratings of willingness to help under major music and minor music conditions.

Figure 4 illustrates participants’ prosocial willingness ratings under major and minor music conditions. There is strong evidence supporting the effect of musical mode and its induced emotions on prosocial decision-making (*t*(27) = 3.273, *p* = 0.003, Cohen’s *d* = 0.619, *BF* = 13.252). Participants in the minor music condition (*M* = 5.89, *SD* = 0.67) exhibited higher prosocial willingness than those in the major music condition (*M* = 5.53, *SD* = 0.88). Correlation analysis showed no significant relationship between willingness to help and empathy scores under either music condition (*N* = 28, *p*s > 0.413).

#### 3.2.3 Mediation analysis

The results for ToM scores showed a trend towards higher ToM scores in the minor music condition (*M* = 5.57, *SD* = 0.63) compared to the happy music condition (*M* = 5.35, *SD* = 0.99), although this difference was not statistically significant (*t*(27) = 1.653, *p* = 0.110, Cohen’s *d* = 0.312, *BF* = 0.669). Additionally, there was no difference in memory strength between the two conditions (*t*(27) = 0.401, *p* = 0.691, Cohen’s *d* = 0.076, *BF* = 0.216).

Mediation analysis indicated a trend towards greater willingness to help in the minor music condition compared to the major music condition (*B* = −0.363, *SE* = 0.199, *p* = 0.069, 95% CI [−0.767, 0.025]). However, neither ToM nor memory strength mediated the relationship between music-induced emotions and prosocial decision-making (indirect effects: ToM, *B* = −0.077, *SE* = 0.079, 95% CI [−0.269, 0.059]; memory strength, *B* = 0.012, *SE* = 0.063, 95% CI [−0.120, 0.160]).

Our findings demonstrate that mode manipulation effectively influences mood rather than arousal. Notably, participants showed a greater willingness to help when exposed to minor music compared to when they listened to major music, indicating that mood changes induced by the music mode can affect prosocial behavior. These findings suggest that the impact of music-induced emotions on prosocial decision-making observed in Experiment 1 can be partially explained by the mood effects of different music modes.

## 4 Experiment 3: effect of tempo-induced emotions on prosocial decision-making

This experiment examined how tempo-induced emotions affect prosocial decision-making. While the tempo information of the musical excerpts from Experiment 1 was retained, the mode was altered. Participants read helping scenario stories while listening to either fast or slow music. Previous research has shown that manipulating tempo affects arousal levels without altering mood (Droit-Volet et al., 2010; Husain et al., 2002). Therefore, it was anticipated that changes in arousal resulting from tempo manipulation might influence participants’ willingness to engage in prosocial behavior.

### 4.1 Materials and methods

#### 4.1.1 Participants

We applied a within-subjects design, in which all participants listened to fast and slow music. *A priori* power analysis using G-power 3.1 (Faul et al., 2009) determined that, to achieve a statistical power of 80% for a paired samples *t*-test detecting a medium effect (*d* = 0.6), at least 24 participants were needed. Consequently, 28 university students (20 females, 8 males) aged 19 to 31 (*M* = 23.36, *SD* = 2.59) participated in the experiment. Aside from general music education in primary and secondary school, none of the participants had received more than 3 years of extracurricular music training. All participants provided informed written consent before the experiment and were compensated for their participation afterward.

#### 4.1.2 Materials and procedure

Similar to Experiment 2, the music stimuli for this experiment consisted of 14 excerpts directly selected from the stimuli provided by Peretz et al. (1998). These excerpts included the seven happy and seven sad pieces used in Experiment 1. For this experiment, the mode of each excerpt was electronically inverted from major to minor and vice versa, while retaining their original tempos. Specifically, the seven happy excerpts were kept at their fast tempos (between 80 and 255 BPM), and the seven sad excerpts were kept at their slow tempos (between 20 and 100 BPM). This resulted in seven fast and seven slow musical excerpts for the experiment, with tempo characteristics preserved despite the mode inversion (see Peretz et al., 1998 for more details). The helping scenario materials, experimental procedure, and statistical methods were consistent with those used in Experiment 2.

### 4.2 Results and discussion

#### 4.2.1 Emotional effects induced by music

**Figure 5:**
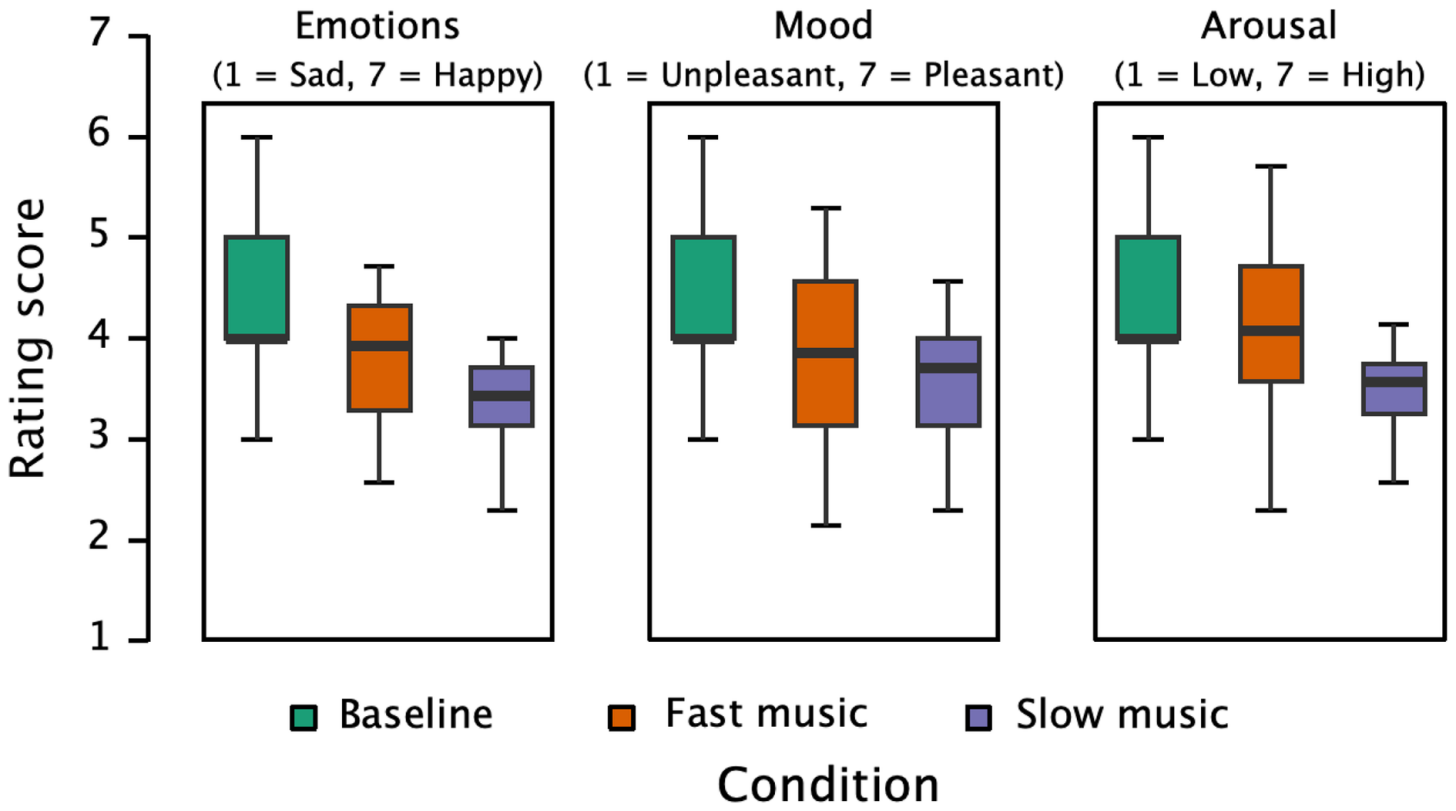
Subjective ratings of emotions (happy/sad), mood, and arousal under baseline, fast music, and slow music conditions.

Figure 5 displays participants’ ratings of happy-sad emotions, mood, and arousal under fast and slow music conditions as well as a baseline condition. There is strong evidence supporting the effect of conditions on happy-sad emotion ratings (*F*(2, 54) = 20.363, *p* < 0.001, partial η^2^ = 0.430, *BF* = 516726.633). Participants’ emotion ratings in the baseline condition (*M* = 4.36, *SD* = 0.73) were higher than those in both the fast music condition (*M* = 3.85, *SD* = 0.62, *p* = 0.004) and the slow music condition (*M* = 3.36, *SD* = 0.47, *p* < 0.001), with ratings in the fast music condition higher than those in the slow music condition (*p* = 0.004).

There is also strong evidence supporting the effect of conditions on mood (*F*(2, 54) = 11.527, *p* < 0.001, partial η^2^ = 0.299, *BF* = 1764.308). Participants’ mood ratings in the baseline condition (*M* = 4.46, *SD* = 0.84) were higher than those in the fast music condition (*M* = 3.77, *SD* = 0.77, *p* < 0.001) and the slow music condition (*M* = 3.56, *SD* = 0.61, *p* = 0.002). However, there was no significant difference in mood ratings between the fast and slow music conditions (*p* = 0.304).

In contrast, there was a significant effect of conditions on arousal ratings (*F*(2, 54) = 13.472, *p* < 0.001, partial η^2^ = 0.333, *BF* = 4194.622). Participants’ arousal ratings in the baseline condition (*M* = 4.32, *SD* = 0.77) were higher than those in the fast music condition (*M* = 4.12, *SD* = 0.81, *p* < 0.001), and the fast music condition had higher arousal ratings than the slow music condition (*M* = 3.45, *SD* = 0.49, *p* < 0.001). This suggests that the manipulation of tempo primarily affects participants’ arousal rather than their mood.

Additionally, post-test results showed that participants preferred listening to slow music (*M* = 4.73, *SD* = 0.77) over fast music (*M* = 4.31, *SD* = 0.72), *t*(27) = 2.318, *p* = 0.028, Cohen’s *d* = 0.438, *BF* = 1.943. Consistent with Experiment 1, all participants reported unfamiliarity with all the music pieces and were unable to correctly identify the titles and composers of the music works during the familiarity assessment.

#### 4.2.2 Effects on prosocial behavior decision-making

**Figure 6:**
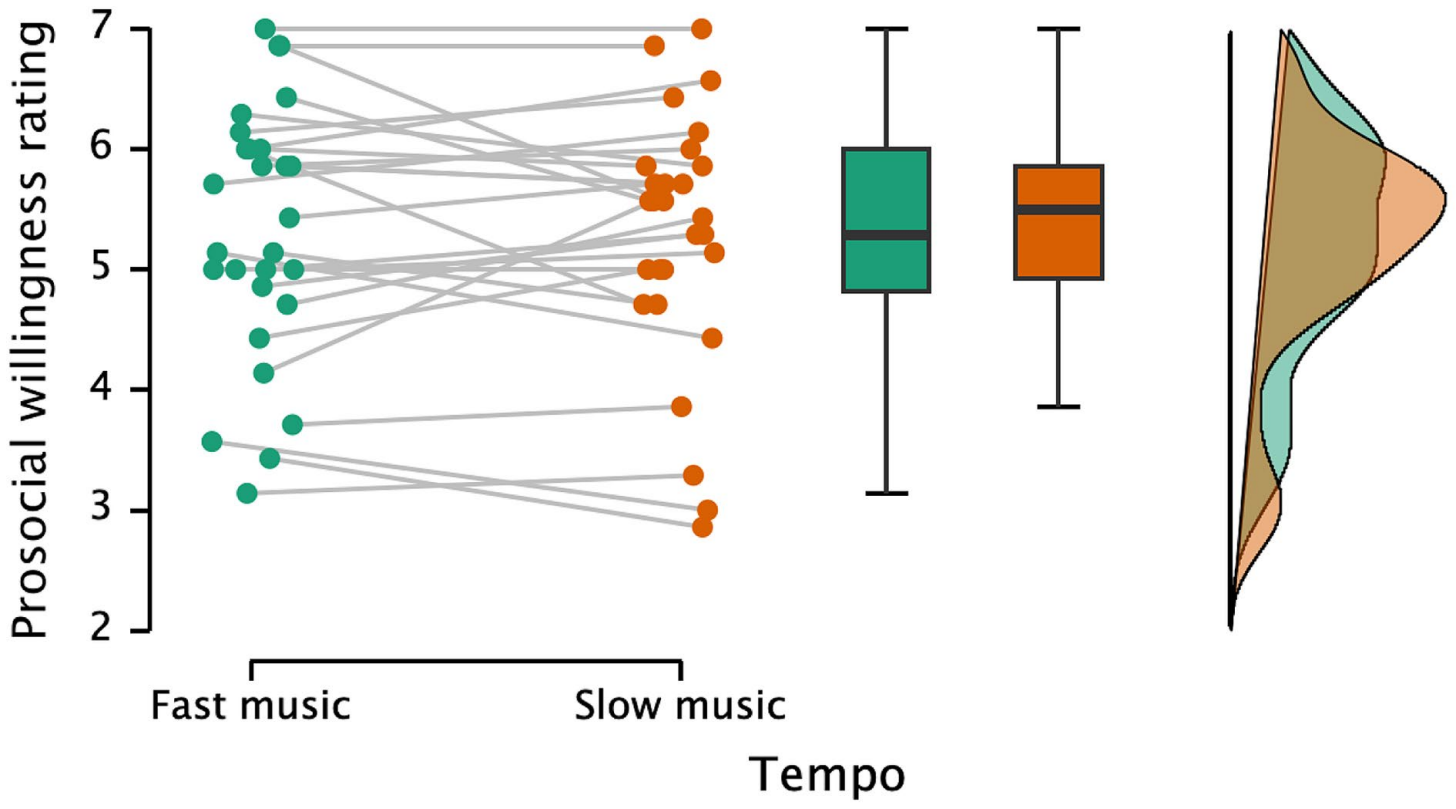
Participants’ ratings of willingness to help under fast music and slow music conditions.

Figure 6 illustrates participants’ prosocial willingness ratings under fast and slow music conditions. There is moderate evidence against the effect of tempo and its induced emotions on prosocial decision-making (*t*(27) = 0.318, *p* = 0.753, Cohen’s *d* = 0.060, *BF* = 0.210), with no difference in prosocial willingness between the fast music condition (*M* = 5.31, *SD* = 1.06) and the slow music condition (*M* = 5.27, *SD* = 1.05).

Furthermore, correlation analysis showed no significant relationship between willingness to help and empathy scores under either music condition (*N* = 28, *p*s > 0.413). Music tempo also did not affect ToM scores (*t*(27) = 1.684, *p* = 0.104, Cohen’s *d* = 0.318, *BF* = 0.698) or memory strength scores (*t*(27) = 0.011, *p* = 0.991, Cohen’s *d* = 0.002, *BF* = 0.201).

The tempo manipulation in this experiment effectively induced changes in arousal without affecting mood. However, arousal changes caused by tempo did not impact prosocial decision-making. These findings suggest that tempo and its arousal effects have less influence on prosocial decisions compared to mode and the associated mood changes.

## 5 General discussion

This study provides novel insights into the influence of music and its induced emotional experiences on prosocial decision-making. Through three behavioral experiments grounded in the two-dimensional model of emotional experience, we systematically examined how different musical characteristics, specifically mode and tempo, affect prosocial decision-making. The findings reveal that while musical mode significantly impacts emotional pleasure and subsequently enhances helping decision-making, tempo primarily influences arousal without affecting prosocial willingness. Furthermore, the mediating roles of ToM and memory strength were insignificant. These results suggest that the structural elements of music, independent of lyrical content, play a critical role in shaping specific emotional experiences that drive prosocial actions, thereby providing empirical support for the music-social bonding hypothesis (Savage et al., 2021).

Overall, the results of the three experiments indicate that sad emotional experiences can enhance prosocial behavior in helping scenarios. This finding aligns with previous research (Lerner et al., 2015; McDonald et al., 2022; Small and Lerner, 2008; Yang et al., 2017), which also highlights the link between sadness and prosocial tendencies. For instance, Yang et al. (2017) demonstrated that participants in a sad condition, induced by autobiographical emotional memory tasks, were more willing to spend time helping others and donate more money compared to those in angry or neutral conditions. The results of the present study further clarified the role of specific emotional dimensions and attributed these effects to distinct musical structural characteristics, thereby expanding upon existing research. However, our findings diverge from some prior studies (e.g., Kniffin et al., 2016; Greitemeyer, 2009a; Skulmowski et al., 2014), possibly due to differences in methodology, such as the type of behavioral decision tasks, the use of music as a prime or background, and the presence of lyrics. In our experiments, participants experienced stronger feelings of sadness and lower pleasure when listening to music, as opposed to baseline conditions. This effect is likely because background music intensified participants’ empathy towards the protagonist’s emotions in the helping scenarios. Compared to happy or major mode music, sad or minor mode music enhanced these emotional experiences, reflecting the impact of state empathy, distinct from trait empathy (Cuff et al., 2016), and may explain the mechanism by which music-induced emotions influence prosocial behavior (Greitemeyer, 2022).

The present study elucidates how specific musical structures or elements can potentially induce stronger prosocial tendencies in individuals. In Experiments 2 and 3, we manipulated musical mode and tempo to examine their respective emotional dimensions’ influence on prosocial decision-making. Our findings align with those from previous studies (e.g., Droit-Volet et al., 2010; Husain et al., 2002; Zhou et al., 2022), demonstrating that mode manipulation influences mood without significantly affecting arousal, while tempo manipulation induces changes in arousal without impacting mood. More importantly, the findings provided strong evidence that only the mode impacted prosocial behavior. However, since this study isolated only the effects of mode and tempo, the conclusions do not imply that other acoustic and structural features of music cannot influence prosocial decision-making. Future research should explore the manipulation of various musical characteristics to further elucidate the relationship between music and prosocial behavior. Interestingly, our previous research found that tempo, rather than mode, affected intertemporal decision-making (Zhou et al., 2022). This suggests that the impact of music on different types of decision-making tasks depends on different acoustic or structural features. Future studies could employ various behavioral decision tasks to explore the specific social functions of distinct musical features.

This study suggests that ToM and cognitive abilities may have a relatively small effects on the prosocial effects of music. Further research in this regard is warranted. Across three experiments, results consistently showed that ToM and memory strength do not influence the impact of music-induced emotions on prosocial decision-making. These findings are in line with previous research by Greitemeyer (2009a) and McDonald et al. (2022). Greitemeyer (2009a) found that while prosocial songs increased prosocial thoughts, this cognitive factor did not translate into prosocial behavior. Similarly, McDonald et al. (2022) noted that emotional music in videos heightened empathy but did not enhance ToM. However, our study diverged from McDonald et al. (2022) by showing that music-induced emotions can affect ToM, though ToM does not mediate the effect of these emotions on prosocial behavior. Given the intricate relationships among empathy, ToM, and cognitive abilities, future research should delve deeper into these connections to better understand their roles in how music influences prosocial decision-making and validate the findings of this study.

One limitation of this study is that it did not directly measure state empathy in the context of music. The present study only measured the affective empathy component of participants’ trait empathy and found no significant correlation between this component and prosocial willingness. Empathy is thought to play a significant role in music-induced emotions, with proposed mechanisms ranging from pre-conscious motor resonance with musical features (Livingstone and Thompson, 2009; Molnar-Szakacs and Overy, 2006) to emotional contagion (Davies, 2011; Juslin and Västfjäll, 2008) and perspective-taking (Levinson, 2006; Scherer and Zentner, 2001). These theories highlight the social significance of music and underscore the need for systematic investigation of empathy-related processes in musical contexts (Clarke et al., 2015). Future research should address this gap by incorporating direct measurements of state empathy to better understand its role in music-influenced prosocial behavior, specifically exploring how background music, whether happy or sad, influences state empathy in helping scenarios. Additionally, future research should explore the relationship between different types of empathy influenced by music and their specific components with prosocial behavior. This investigation could provide deeper insights into how various forms of empathy—such as affective empathy, cognitive empathy, and state versus trait empathy—contribute to prosocial actions when induced by music. Understanding these nuances may help elucidate the mechanisms through which music-induced empathy fosters helping behaviors and identify which aspects of empathy are most influential in promoting prosociality. In retrospect, another potential limitation of the current experiments was the reliance on within-subjects rather than between-subjects designs. As participants were exposed to both happy and sad music while reading the narratives, there is a possibility that they inferred the purpose of the experiment, which might have introduced demand characteristics that influenced their responses. This awareness could have unintentionally impacted the validity of the findings. To reduce such risks in future research, adopting a between-subjects design could minimize the likelihood of participants forming hypotheses about the study’s objectives.

It is also important to acknowledge that while our study focused on isolating the effects of mode and tempo, this approach may have inadvertently simplified the emotional richness of the music. By using ‘major/fast’ versus ‘minor/slow’ manipulations and relying on MIDI-based stimuli instead of sampled audio tracks, we aimed to eliminate the influence of performative expressiveness. However, this simplification could overlook other acoustic and structural factors that contribute to the full complexity of emotional responses in music. For instance, intrinsic characteristics of the compositions (e.g., pitch height, interval size, tonality, harmony, melodic contour, rhythm, and musical form) and performative features (e.g., agogics, phrasing, articulation, and timbre) can significantly influence emotional expression and perception (Gabrielsson and Lindström, 2010; Huron, 2008; Juslin and Timmers, 2010), and these elements are often interrelated (e.g., Huron and Royal, 1996; Schutz, 2017). For example, sad expressions in musical performance are typically associated with low sound levels, legato articulation, minimal articulation variability, slow tone attacks, and soft timbre, while happy expressions are linked to high sound levels, staccato articulation, greater articulation variability, fast tone attacks, and bright timbre (Juslin, 1997, 2000; Juslin and Timmers, 2010). Additionally, affective pianistic performances tend to exhibit more agogics, legato, and dynamic variation compared to more cognitively focused performances (Higuchi et al., 2011). Future research should incorporate these finer musical characteristics and use more ecologically valid stimuli to better capture the complexity of emotional responses to music.

Furthermore, the effects observed in the present study are primarily based on the characteristics of Western music and listeners familiar with Western musical traditions, reflecting a Eurocentric premise that may limit the generalizability of the findings across different cultural contexts. It is well-established that the perception and experience of musical emotions are deeply influenced by cultural context. Indeed, even Kivy (1981) acknowledged that each musical culture has its own distinct principles and practices that influence how music is perceived emotionally. While research has demonstrated a degree of universality in the processing of basic emotions across different musical cultures (Balkwill, 2006; Balkwill and Thompson, 1999; Balkwill et al., 2004; Fritz et al., 2009), the specific acoustic cues that listeners rely on can vary significantly depending on their cultural background (Zacharopoulou and Kyriakidou, 2009). For instance, in Balkwill’s (2006) study, Canadian and Japanese listeners were both able to interpret emotions in Japanese, Hindustani, and Western music, but they relied on different acoustic cues for certain emotions. Canadians primarily used intensity to judge anger, whereas Japanese listeners considered complexity, tempo, and intensity. These differences may stem from variations in cognitive styles or attentional focus shaped by cultural contexts (Nisbett et al., 2001). Moreover, Egermann et al. (2015) found that while arousal responses to music are consistent across cultures, emotional valence is more likely to be shaped by cultural influences. Consequently, listeners from different cultural backgrounds may process musical emotions using different cues, and as cultural adaptation occurs, reliance on culturally specific cues may increase (Thompson and Balkwill, 2010). Future research should therefore consider the cultural specificity of musical emotion processing to better understand how varying cultural perspectives among participants may influence the emotional and social functions of music.

## 6 Conclusion

In conclusion, this study highlights the significant impact of musical mode on emotional pleasure and prosocial decision-making, while demonstrating that tempo influences arousal without affecting prosocial willingness. These findings underscore the importance of specific musical structural characteristics in shaping emotional experiences that drive prosocial behavior. Future research should continue investigating the complex relationships among empathy, ToM, cognitive abilities, and their roles in how music influences prosocial behavior. Additionally, further exploration of various musical features using more ecologically valid stimuli and considering cultural differences will enhance our understanding of how music affects human behavior.

## Data Availability

The raw data supporting the conclusions of this article will be made available by the authors, without undue reservation.

## Supplementary material

Supplementary Table 1 Detailed information about the music pieces used in this study.

Supplementary Table 2 Detailed information about the story materials used in this study.
