# Supplementary material for: Tunes that move us: the impact of music-induced emotions on prosocial decision-making
Source: Front Psychol. 2025 Jan 9;15:1453808. doi: 10.3389/fpsyg.2024.1453808 (PMC11754231; doi:10.3389/fpsyg.2024.1453808)
Supplement: Supplementary Table 1 — Detailed information about the music pieces used in this study. [file Table_1.DOCX]

Supplementary Material

**Supplementary Table 1.** Detailed information about the music pieces used in this study.

| Composer | Work | Measure | Tempo | Tonality | Emotion |
| --- | --- | --- | --- | --- | --- |
| Haendel | Utrecht’s Te Deum | 5–14 | 112 | D Major | Happy |
| Saint-Saëns | Carnaval des Animaux (Finale) | 10–26 | 220 | C Major | Happy |
| Saint-Saëns | Carnaval des Animaux (La volière) | 1–9 | 88 | F Major | Happy |
| Mozart | Eine kleine nachtmusik (1st mvt) | 5-10 | 154 | G Major | Happy |
| Mozart | Piano Concerto no. 23 (3rd mvt) | 1–8 | 255 | A Major | Happy |
| Mozart | Piano Concerto no. 27 (3rd mvt) | 1–8 | 167 | B-ﬂat Major | Happy |
| Verdi | Rigoletto (Act 1 no. 4) | 69–73 | 150 | C Major | Happy |
| Albinoni | Adagio | 7–14 | 48 | G Minor | Sad |
| Bruch | Kol Nidrei | 9–11 | 20 | D Minor | Sad |
| Chopin | Nocture Op 48 no. 1 | 1–4 | 52 | C Minor | Sad |
| Debussy | Prélude: Des pas sur la Neige | 4–8 | 35 | D Minor | Sad |
| Mahler | Symphony no. 5 (3rd mvt) | 12–16 | 54 | A Minor | Sad |
| Mozart | Piano Concerto no. 23 (2nd mvt) | 1–3 | 35 | F-sharp Minor | Sad |
| Schubert | String Quartet no. 14 (2nd mvt) | 1–4 | 72 | G Minor | Sad |
