# Supplementary material for: Tunes that move us: the impact of music-induced emotions on prosocial decision-making
Source: Front Psychol. 2025 Jan 9;15:1453808. doi: 10.3389/fpsyg.2024.1453808 (PMC11754231; doi:10.3389/fpsyg.2024.1453808)
Supplement: Supplementary Table 2 — Detailed information about the story materials used in this study. [file Table_2.DOCX]

Supplementary Material

**Supplementary Table 2.** Detailed information about the story materials used in this study.

| No. | Story Text (in Chinese) | Translation (in English) |
| --- | --- | --- |
| 01 | 下班回家后，张明照例带着小狗旺旺出门遛弯。一走出家门，张明用力朝远方的小山丘抛出一个球。旺旺和往常一样飞奔而出，追向小山丘方向。张明站在家门口等待，没成想，许久都不见旺旺归来。沿着小山丘搜寻许久，张明没找到旺旺，也没找到球。24个小时过去了，旺旺还没有回家。 | After getting off work and going home, Zhang Ming habitually took his dog, Wangwang, out for a walk. As soon as he stepped out of the house, Zhang Ming threw a ball forcefully toward a small hill in the distance. As usual, Wangwang dashed off, running towards the hill. Zhang Ming stood at the door, waiting. Unexpectedly, Wangwang did not return for a long time. After searching around the small hill for a while, Zhang Ming couldn't find Wangwang, nor the ball. Twenty-four hours passed, and Wangwang still hadn't come home. |
| 02 | 炎热的周六上午，刘尚被闹铃吵醒。他拿起床头的手机关掉闹铃，正好来了通电话。刘尚接通电话，是修空调的王师傅。王师傅已经到刘尚的小区门口了，需要刘尚过去引路。刘尚起身换上衣服，匆匆出门跑向小区大门。接上了王师傅，刘尚简单描述了空调的运行情况。刘尚摸了摸口袋，意识到没带钥匙。 | On a hot Saturday morning, Liu Shang was awakened by his alarm clock. He picked up his phone from the bedside table to turn off the alarm, and a call came in just at that moment. Liu Shang answered the call; it was Master Wang, the air conditioner repairman. Master Wang had already arrived at the entrance of Liu Shang's residential complex and needed Liu Shang to come and guide him. Liu Shang got up, changed his clothes, and hurriedly ran to the entrance of the complex. After picking up Master Wang, Liu Shang briefly described the air conditioner's performance. Liu Shang patted his pockets and realized he hadn't brought his keys. |
| 03 | 周五下午，轮到姜岩做值日。他把一个个课桌排列整齐，随后又拿起扫帚，打扫起地板上的垃圾。清扫干净后，姜岩把簸箕里的垃圾倒入垃圾桶里，并给垃圾桶换上了新的垃圾袋。随后，他拖着一袋满满的垃圾，往垃圾站走去。走到半截，垃圾袋破了，手也被碎玻璃划出了血。 | On Friday afternoon, it was Jiang Yan's turn to be on duty. He arranged the desks neatly one by one, then picked up a broom and started sweeping the trash off the floor. After cleaning up, Jiang Yan emptied the dustpan into the trash bin and replaced the bin with a new bag. Then, he dragged a bag full of garbage towards the trash station. Halfway there, the garbage bag broke, and his hand was cut by broken glass, causing it to bleed. |
| 04 | 星期六的晚上，方嘉和爸爸一起去逛商场。一开始，爸爸牵着她的手。路过一个数码店，爸爸停下来挑选手机，让方嘉在一旁等他。方嘉站在旁边，环顾四周，看见对面有一个玩具店。各式各样的娃娃吸引了她，她忘记了爸爸的嘱咐，跑到玩具店里。再回头时，找不到爸爸了。 | On Saturday evening, Fang Jia went shopping at the mall with her dad. At first, her dad held her hand. When they passed by a digital store, her dad stopped to look at some phones and told Fang Jia to wait beside him. Fang Jia stood nearby, looking around, and saw a toy store across the way. The various dolls caught her attention, and she forgot her dad's instructions and ran into the toy store. When she turned back, she couldn't find her dad. |
| 05 | 今天早上，赵爱国在小区里散步。他停留在老年健身器械处，似乎准备活络一下筋骨。反常的是，他愣了一会神，然后往小区门口走去。他一边走一边念叨着些什么，不知不觉，走进了附近的菜市场。他买了三个番茄，付完钱后忘了拿，又买了一条鱼。他在鱼摊前，忘记了回家的路。 | This morning, Zhao Aiguo was taking a walk in his neighborhood. He stopped at the exercise equipment for seniors, seemingly ready to do some stretches. Strangely, he stood there in a daze for a while, then started walking towards the neighborhood entrance. Muttering to himself as he walked, he unwittingly wandered into a nearby vegetable market. He bought three tomatoes but forgot to take them after paying, then bought a fish. At the fish stall, he forgot the way back home. |
| 06 | 阳光明媚的周末，钱强给花园里的花浇水。他照着网上的方法，先浇灌花的根部，再用水管大面积喷洒。突然，耳边传来几阵“嗡嗡”声，飞来了几只蜜蜂。钱强将水管换了个方向，别过头去，不停的摇头。蜜蜂还是蜇到了他的手。一转头，远处又有几只蜜蜂飞过来了。 | On a sunny weekend, Qian Qiang was watering the flowers in his garden. Following instructions he found online, he first watered the roots of the flowers and then used a hose to spray a wider area. Suddenly, he heard several buzzing sounds as a few bees flew over. Qian Qiang redirected the hose and turned his head away, shaking it continuously. Despite his efforts, a bee stung his hand. When he looked around, he saw a few more bees flying towards him from a distance. |
| 07 | 新一周的清晨，林爱华拿着上课用的教材出门了。走到家门口，他刷了一辆共享单车。林爱华把上课用的教材放入车篓中，检查了一下车子，往学校骑去。到了学校门口，他看了眼手表，匆忙锁上车就走了。在教室坐下后，他收拾了一下桌面，想起来教材落在车篓里了。此时，上课铃响了。 | On a new week's morning, Lin Aihua left home with his teaching materials. At his doorstep, he scanned a shared bike. Lin Aihua placed the teaching materials in the bike's basket, checked the bike, and rode towards the school. Upon reaching the school gate, he glanced at his watch, quickly locked the bike, and hurried off. After sitting down in the classroom, he tidied up his desk and realized that he had left the teaching materials in the bike's basket. At that moment, the class bell rang. |
| 08 | 八月的最后一天，杨蕾坐高铁去学校。上车后，她找到靠窗的座位坐下了。为了打发漫长的乘车时间，她拿出蓝牙耳机，开始听音乐。不知不觉，还有10分钟就到站了。她动了动身子，一转身，耳机掉在了座椅的缝隙里。她伸手去够，奈何手太短，伸不进去。 | On the last day of August, Yang Lei took the high-speed train to school. After boarding, she found a window seat and sat down. To pass the long travel time, she took out her Bluetooth earphones and started listening to music. Before she knew it, there were only 10 minutes left until her stop. She shifted in her seat, and as she turned around, her earphones fell into the gap between the seats. She reached in to retrieve them, but her hand was too short to reach. |
| 09 | 周五晚上，王妍加完班后准备回家。她赶往公交车站，搭乘上了最后一班18路公交车。她走上公交车，量了体温后，从包里掏出手机。她打开电子乘车卡的页面，此时，手机自动关机了。王妍重启手机也没反应，她到包里翻了翻，也没有零钱。她站在前边车门处，不知如何是好。 | On Friday night, after working overtime, Wang Yan was ready to go home. She hurried to the bus stop and caught the last Route 18 bus. After boarding, she had her temperature checked and pulled her phone out of her bag. Just as she opened the electronic bus card page, her phone suddenly powered off. Wang Yan tried to restart her phone, but it didn't respond. She searched through her bag but couldn't find any loose change. She stood by the front door of the bus, not knowing what to do. |
| 10 | 上完最后一节课后，李玉踏着放学铃走出了校园。她走向自行车棚，找到了自己的自行车。她把书包放在了篓子里，给车解了锁，便骑往家的方向。骑到半截，书包带子卷进了李玉的车轮子里。李玉蹬了蹬脚蹬，用不上劲，感觉怎么也蹬不动。她停下自行车检查，原来是车链子掉了。 | After finishing her last class, Li Yu walked out of the school as the dismissal bell rang. She headed to the bike shed and found her bicycle. She placed her backpack in the basket, unlocked the bike, and started riding home. Midway, the strap of her backpack got caught in the bike wheel. Li Yu tried to pedal, but she couldn't apply any force and felt the bike wouldn't move. She stopped to check and found that the chain had come off. |
| 11 | 下课后，孙芝去图书馆学习。她先把电脑放下，然后去书架找书。不知不觉，已经10点了，图书馆响起了闭馆铃。孙芝收拾了一下东西，去借书处借了要用的书，之后走出图书馆。外面黑漆漆一片，突然，下起了瓢泼大雨。因为没带伞，孙芝只能躲在图书馆门口的屋檐下。 | After class, Sun Zhi went to the library to study. She first set down her computer and then went to the shelves to find some books. Before she knew it, it was already 10 o'clock, and the library's closing bell rang. Sun Zhi gathered her things, borrowed the books she needed, and walked out of the library. Outside, it was pitch dark, and suddenly, it started pouring rain. Since she didn't have an umbrella, Sun Zhi could only take shelter under the eaves at the library entrance. |
| 12 | 今天中午，何慧去食堂吃饭。她走进食堂，看见了食堂被拖的锃亮的地。她走到打饭窗口前，打了两荤一素，随后刷了饭卡。孙慧拿上了打好的饭，排在打汤的队伍后，准备拿汤。她小心翼翼的把汤碗放在饭盘上，一转身，一个踉跄就滑倒了。何慧的身上洒满了饭，手臂也摔了。 | Today at noon, He Hui went to the cafeteria for lunch. She walked into the cafeteria and noticed the freshly mopped, gleaming floor. She went to the food counter and got two meat dishes and one vegetable dish, then swiped her meal card. Sun Hui took her meal and stood in line for soup. She carefully placed the soup bowl on her tray, but as she turned around, she slipped and fell. He Hui spilled her meal all over herself and hurt her arm. |
| 13 | 春光明媚，郑雯去郊区爬山。一开始山路较缓，温度也适宜。她一鼓作气，一下也没休息，很快便到达了半山腰。她找了一个亭子，拿出一些食物，休息了一会儿。越往上爬，温度越低，台阶上布满了青苔。一不小心，她扭到了脚。 | On a bright spring day, Zheng Wen went hiking in the suburbs. Initially, the mountain path was gentle, and the temperature was pleasant. She kept going without taking a break and soon reached the halfway point. She found a pavilion, took out some food, and rested for a while. As she climbed higher, the temperature dropped, and the steps were covered in moss. She accidentally twisted her ankle. |
| 14 | 秋高气爽，倪芳将家中新鲜的水果搬到三轮车上准备出摊。她把一箱箱的水果堆在三轮车里，并用绳子固定住。刚出发，便飘起了绵绵细雨，路上也堵满了车。倪芳加速着前行，心想找到一个好的摊位，早一些收工。骑到十字路口时，她一个急刹，车上的橘子滚落了满地。她下车查看，原来是后侧的护栏垮掉了。 | On a crisp autumn day, Ni Fang loaded fresh fruits from her home onto a tricycle to set up her stall. She piled boxes of fruit onto the tricycle and secured them with ropes. Just as she set off, a light drizzle began to fall, and the roads were crowded with cars. Ni Fang accelerated, hoping to find a good spot and finish early. As she reached an intersection, she braked suddenly, and the oranges rolled all over the ground. She got off to check and found that the rear side rail had collapsed. |
| 15 | 周一上午，陈娟像往常一样去公司上班。她把包放在工位上，去休息处泡咖啡。这时，电话铃响起，陈娟四处找她的手机。她在办公椅上找到了手机，匆忙接通了电话，边说边在桌上找文件。手忙脚乱之下，她把咖啡打翻了，咖啡液洒的电脑和地上到处都是。她又开始找纸和抹布，没有找到。 | On Monday morning, Chen Juan went to work as usual. She placed her bag at her workstation and went to the break area to make some coffee. Just then, her phone rang, and Chen Juan looked around for it. She found the phone on her office chair and hurriedly answered the call while looking for some documents on her desk. In the rush, she accidentally knocked over her coffee, spilling it all over her computer and the floor. She then started looking for paper towels and a cloth but couldn't find any. |
| 16 | 一个下雨天，高红去快递柜拿快递。正逢双十一购物节，她买了一些日用品。刚取出一个快递，正在这时，又有一个快递到了。她把取好的快递放在地上，扫描取件二维码，取出另一个快递。紧接着，她撑开伞，试图抱起两个快递。然而，快递体积太大了她拿不下。 | On a rainy day, Gao Hong went to the parcel locker to pick up her packages. It was during the Double Eleven shopping festival, and she had bought some daily necessities. Just as she took out one package, another one arrived. She placed the first package on the ground, scanned the pickup QR code, and retrieved the second package. Then, she opened her umbrella and tried to carry both packages. However, the packages were too large for her to hold. |
| 17 | 新学期开始，袁媛去学校报到。她拎着大包小包，朝宿舍走去。此时，天空下起了雨，袁媛停住了脚步。她把书包放在行李箱上，拿出雨伞，并腾出了一只手撑伞。她一手撑着伞，一手拖着行李箱和几袋生活用品，步履维艰地向前走。一不注意，行李箱侧翻了，一堆东西从袋子里滚出来，散落在地上。 | At the start of the new semester, Yuan Yuan went to school to register. Carrying bags and suitcases, she headed towards the dormitory. At that moment, it began to rain, and Yuan Yuan stopped in her tracks. She placed her backpack on the suitcase, took out her umbrella, and freed one hand to hold it. With one hand holding the umbrella and the other dragging the suitcase and several bags of daily necessities, she struggled forward. Not paying attention, her suitcase tipped over, and a bunch of items rolled out of the bags, scattering all over the ground. |
| 18 | 周三上午，薛婕带着她两岁的儿子浩浩去看牙。她抱着睡眼朦胧的浩浩，走出家门，赶往公交车站。正值早高峰时间段，公交车站挤满了人，薛婕找了一个公交站牌靠着。不一会儿，去医院的车来了，她在人群里缓缓走上车。公交车厢里坐满了人，浩浩被挤的哭了起来。 | On Wednesday morning, Xue Jie took her two-year-old son, Haohao, to the dentist. She carried the sleepy Haohao out of the house and hurried to the bus stop. It was rush hour, and the bus stop was crowded with people. Xue Jie found a spot to lean against the bus stop sign. Soon, the bus to the hospital arrived, and she slowly made her way onto the bus through the crowd. The bus was packed, and Haohao started crying from being squeezed. |
| 19 | 6月的最后一天，宋青拖着一堆行李去车站坐车。她早早的进了站，等待检票。不一会儿，列车开始检票，她拖着行李上了车。她进了自己的车厢，找到自己的位置，把背包放在了座位上。紧接着，她站回过道里，看了看头顶的行李架。她试图把行李箱放上去，然而提起来又放下了。 | On the last day of June, Song Qing dragged a pile of luggage to the station to catch a train. She arrived early and waited for the ticket check. Before long, the train began boarding, and she hauled her luggage onto the train. She entered her carriage, found her seat, and placed her backpack on it. Then she stood back in the aisle and looked at the overhead luggage rack. She tried to lift her suitcase onto the rack but ended up putting it down again. |
| 20 | 七月的一个上午，黄鑫出门送外卖。他早早的出了门，心想多接几单。他骑着电动车，到餐馆里，等待商家做好饭。没过多久，商家打包好了饭菜，黄鑫将其放入外卖箱中。他骑上电动车，开往外卖送达地。此时已到正午，他感觉头脑发昏，停下车靠在路边的树上。 | On a July morning, Huang Xin went out to deliver food. He left early, intending to take more orders. Riding his electric scooter, he arrived at the restaurant and waited for the food to be prepared. Before long, the restaurant had the meals packed, and Huang Xin placed them in his delivery box. He got back on his scooter and headed towards the delivery location. By noon, he started to feel dizzy and stopped by the roadside, leaning against a tree. |
| 21 | 又到了美术课，尹晓走到教室。她找到自己的座位坐下，把工具箱放在桌上。“叮铃铃”，上课铃响了，老师开始讲课。认真听完老师的要求后，她打开工具箱，准备开始画画。她找遍了工具箱，都没有找到水彩笔。她回想了一下，原来是昨晚做完美术作业后落在家里了。 | It was time for art class again, and Yin Xiao walked into the classroom. She found her seat, sat down, and placed her toolbox on the desk. "Ring, ring," the class bell rang, and the teacher began the lesson. After carefully listening to the teacher's instructions, she opened her toolbox, ready to start painting. She searched through the toolbox but couldn't find her watercolor pens. Thinking back, she realized she had left them at home after finishing her art homework the previous night. |
